# Long-term effects of an inpatient weight-loss program in obese children and the role of genetic predisposition-rationale and design of the LOGIC-trial

**DOI:** 10.1186/1471-2431-12-30

**Published:** 2012-03-19

**Authors:** Melanie Rank, Monika Siegrist, Désirée C Wilks, Bernhard Haller, Bernd Wolfarth, Helmut Langhof, Martin Halle

**Affiliations:** 1 Department of Prevention, Rehabilitation and Sports Medicine, Technische Universität München, Klinikum rechts der Isar, Munich, Germany; 2 Institute for Medical Statistics and Epidemiology, Technische Universität München, Klinikum rechts der Isar, Munich, Germany; 3 Rehabilitation Clinic, Klinik Schönsicht', Berchtesgaden, Germany; 4 Munich Heart Alliance, Munich, Germany

**Keywords:** Lifestyle intervention, Polymorphism, Follow-up, Adipokines, Inflammation, Fitness

## Abstract

**Background:**

The prevalence of childhood obesity has increased worldwide, which is a serious concern as obesity is associated with many negative immediate and long-term health consequences. Therefore, the treatment of overweight and obesity in children and adolescents is strongly recommended. Inpatient weight-loss programs have shown to be effective particularly regarding short-term weight-loss, whilst little is known both on the long-term effects of this treatment and the determinants of successful weight-loss and subsequent weight maintenance.

The purpose of this study is to evaluate the short, middle and long-term effects of an inpatient weight-loss program for children and adolescents and to investigate the likely determinants of weight changes, whereby the primary focus lies on the potential role of differences in polymorphisms of adiposity-relevant genes.

**Methods/Design:**

The study involves overweight and obese children and adolescents aged 6 to 19 years, who participate in an inpatient weight-loss program for 4 to 6 weeks. It started in 2006 and it is planned to include 1,500 participants by 2013. The intervention focuses on diet, physical activity and behavior therapy. Measurements are taken at the start and the end of the intervention and comprise blood analyses (DNA, lipid and glucose metabolism, adipokines and inflammatory markers), anthropometry (body weight, height and waist circumference), blood pressure, pubertal stage, and exercise capacity. Physical activity, dietary habits, quality of life, and family background are assessed by questionnaires. Follow-up assessments are performed 6 months, 1, 2, 5 and 10 years after the intervention: Children will complete the same questionnaires at all time points and visit their general practitioner for examination of anthropometric parameters, blood pressure and assessment of pubertal stage. At the 5 and 10 year follow-ups, blood parameters and exercise capacity will be additionally measured.

**Discussion:**

Apart from illustrating the short, middle and long-term effects of an inpatient weight-loss program, this study will contribute to a better understanding of inter-individual differences in the regulation of body weight, taking into account the role of genetic predisposition and lifestyle factors.

**Trial Registration:**

NCT01067157.

## Background

The global increase in childhood overweight and obesity is a serious health concern [1], as it often tracks into adulthood [2] where it is associated with numerous cardiovascular and metabolic risk factors such as hypertension, type 2 diabetes or hyperlipidemia and even cardiovascular disease [3,4]. In addition, even at young age, overweight and obesity are related with various physical and psychological comorbidities. For instance, it has been found that overweight and obese children and adolescents often suffer from elevated blood pressure, dyslipidemia or disorders of glucose metabolism [5], and have a lower quality of life compared to healthy normal weight children [6].

### Obesity and inflammation

The link between adiposity and the development of metabolic and cardiovascular diseases may be seen in obesity-related systemic inflammation [7,8]. Hypertrophy and hyperplasia of the adipose tissue as seen in obesity result in a dysfunction of the adipocytes [9], which increases inflammation and impairs hemostasis, glucose as well as lipid metabolism [7,8]. This is triggered by an alteration of the secretion of the adipokines adiponectin, leptin, retinol binding protein 4 (RBP4) and resistin as well as inflammatory markers such as interleukin 6 (IL-6), tumor necrosis factor-α (TNF-α) and C-reactive protein (CRP). For example, a decrease in adiponectin and an increase of RBP4 as often found in obese individuals may foster the development of insulin resistance. Furthermore, elevated levels of RBP4, IL-6 and TNF-α increase the inflammatory status by directly stimulating CRP synthesis in the liver [9].

In contrast, physical activity and/or weight-loss seem to have a positive impact on these mechanisms by improving the inflammatory status and reducing insulin resistance. However, data concerning these mechanisms in children are scarce and results from the existing studies have been inconsistent [9,10]. In addition, simultaneous measurements of adipokines, inflammatory markers, and cardiovascular risk factors of obese children before and after a short-term lifestyle intervention and at a long-term follow-up during late adolescence or adulthood have not been performed before.

### The role of genes

Weight gain due to an increase in adipose tissue is the result of an imbalance between energy expenditure and energy intake. This balance can be influenced by both physical activity and caloric intake, which can be dependent on social, psychological and other behavioral factors. In addition, genes have been shown to play a fundamental role in the regulation of body weight [1,11]. Apart from very rare monogenetic disorders [12], a genetically determined higher risk for obesity can often be attributed to a polygenetic pattern involving different single nucleotide polymorphisms (SNP's). For instance, variations in the *FTO*-gene seem to have an effect on the development of early onset obesity. Likewise, a study by Frayling et al. has shown that a single-nucleotide polymorphism of the SNP *rs9939609*A allele is associated with an increased risk of overweight (odds ratio 1.18; 95% CI = 1.13 to 1.24) and obesity (odds ratio 1.31; 95% CI = 1.23 to 1.39), increasing the risk by 20-30%. Additionally, the A allele of the *rs9939609 SNP *has been found to be associated with an increased body mass index (BMI) in 7 year old children and to also determine obesity during puberty and beyond [13]. Furthermore loci associated with neuronal pathways (*TMEM18, GNPDA2, SH2B1, NEGR1*) have recently been identified to be associated with childhood obesity [14]. It has to be noted though that these genetic predispositions may only lead to an obesity phenotype in the presence of an obesogenic environment, and therefore this association may be modified by a lifestyle intervention [15,16].

### Lifestyle interventions to treat childhood obesity

Due to the tremendous short and long-term health consequences, current recommendations strongly encourage the treatment of childhood obesity, which may be performed in an outpatient or an inpatient setting (e.g. residential or weight-loss camps), or by a combination of both. However, the effectiveness of these types of programs remains uncertain [17]. In a recent review by Kelly and Kirschenbaum the average decrease in percent overweight within inpatient treatment across 11 studies was reported 23.9% from pre to post-intervention and 20.6% from pre-intervention to follow-up, whereas the effect on percent overweight was 8.5% and 8.9% for outpatient programs, respectively [18]. Within the EvAKuJ-study (Evaluation of obesity treatment in children and adolescents study) the short and long-term effects of different German childhood-obesity programs were assessed [19]. The authors reported that five out of 48 programs included took place in an inpatient setting (875 patients), whereas all others were carried out in an outpatient setting (1,041 patients). Children participating in inpatient programs achieved a mean reduction in BMI-SDS (BMI standard deviation score [20,21]) of -0.36 during the treatment and of -0.17 during the observational follow-up 1-2 years after termination of the treatment, whereas this was -0.18 and -0.21 for outpatient programs, respectively [19]. In summary, the results of inpatient versus outpatient programs are equivocal especially regarding long-term effectiveness.

Furthermore, as presented above, very few inpatient treatment programs have been evaluated, and these studies are heterogeneous regarding their study design and overall quality. For instance, the treatment duration ranges from 10 days to 10 months and only 29% of the studies included a follow-up period. The range in follow-up duration also varies dramatically (4 months to 4.6 years) and about half (46%) of the studies performed a follow-up after less than 1 year [18,22,23]. A study by Braet and van Winckel is the only one with a follow-up period of more than 3 years from the start of the intervention, however, they have not carried out blood analyses and the sample size of their inpatient treatment group was rather low [24].

These results emphasize that inpatient treatment might be the most effective strategy for children to loose body weight in the short-term, but that there is a substantial need for intervention studies with considerably longer duration of follow-up and a standardized protocol of the intervention and analyses. In addition, only very few studies have reported on the influence of lifestyle intervention in obese children whilst considering genetic predisposition [25-30].

## Methods/Design

### Objectives

To investigate the determinants for short, middle and long-term weight-loss and weight maintenance, a prospective cohort study involving overweight and obese children and adolescents (hereafter referred to as 'children') is being conducted, which includes a short-term inpatient weight-loss program complemented by a long-term observational follow-up over 10 years. Measurements include anthropometric, cardiometabolic and genetic parameters as well as assessment of physical activity and fitness, dietary habits and quality of life.

#### Primary endpoint

The associations between polymorphisms in adiposity-relevant genes (e.g. *FTO, MC4R, TMEM-18*) on the changes in BMI and BMI-SDS after a controlled lifestyle intervention (4 to 6 weeks) in overweight and obese children and adolescents.

#### Secondary endpoints

The short (4 to 6 weeks), middle (6 to 12 months) and long-term (2, 5 and 10 years) effects of the intervention on the below-listed parameters and their associations with polymorphisms in adiposity-relevant genes (*e.g. FTO, MC4R, TMEM-18*):

• anthropometric parameters

• parameters of lipid and glucose metabolism

• adipokines and inflammatory markers

• physical fitness

• physical activity

• dietary behavior and intake

• health-related quality of life

### Participants

Participants of the LOGIC-trial (**L**ong-term effects of lifestyle intervention in **O**besity and **G**enetic **I**nfluence in **C**hildren) are 6 to 19 year old overweight and obese children, who are referred to the rehabilitation center *Klinik Schönischt *in Berchtesgaden, Germany by their local pediatrician to have inpatient weight-loss treatment. The clinic is specialized on childhood obesity and about 200 children with the primary diagnosis overweight/obesity are being treated here annually.

Children are admitted to the clinic on a biweekly basis and recruited consecutively by scientists from the *Department of Prevention, Rehabilitation and Sports Medicine, Technical University of Munich*. In case they fulfill the inclusion criteria (see Table 1), assent and informed consent for study participation are obtained from the children and their accompanying legal guardians.

**Table 1:** Inclusion and exclusion criteria for participation in the LOGIC-trial

	Inclusion criteria	Exclusion criteria
**Eligibility criteria for attending the inpatient weight-loss program at the *Klinik Schönsicht***	Overweight (BMI 90.-97^th ^percentile), obese (BMI 97.-99.5^th ^percentile) or severely obese (BMI > 99.5^th ^percentile) Repeated failure to accomplish weight-loss in outpatient therapies	Considerable mental or physical disability Severe personality disorders Suicidal behavior Drug addiction

**Eligibility criteria for LOGIC-trial participation**	Written informed consent by participant and a legal guardian	Obesogenic diseases and disorders such as the Prader-Willi Syndrome, Cushing Syndrome Early withdrawal from the inpatient program (< 3 weeks)

The study is conducted according to the declaration of Helsinki (Seoul, 2008) and approved by the ethics committee of the Faculty of Medicine of the Technische Universität München, Germany (1354/05).

### Recruitment process

**Figure 1:**
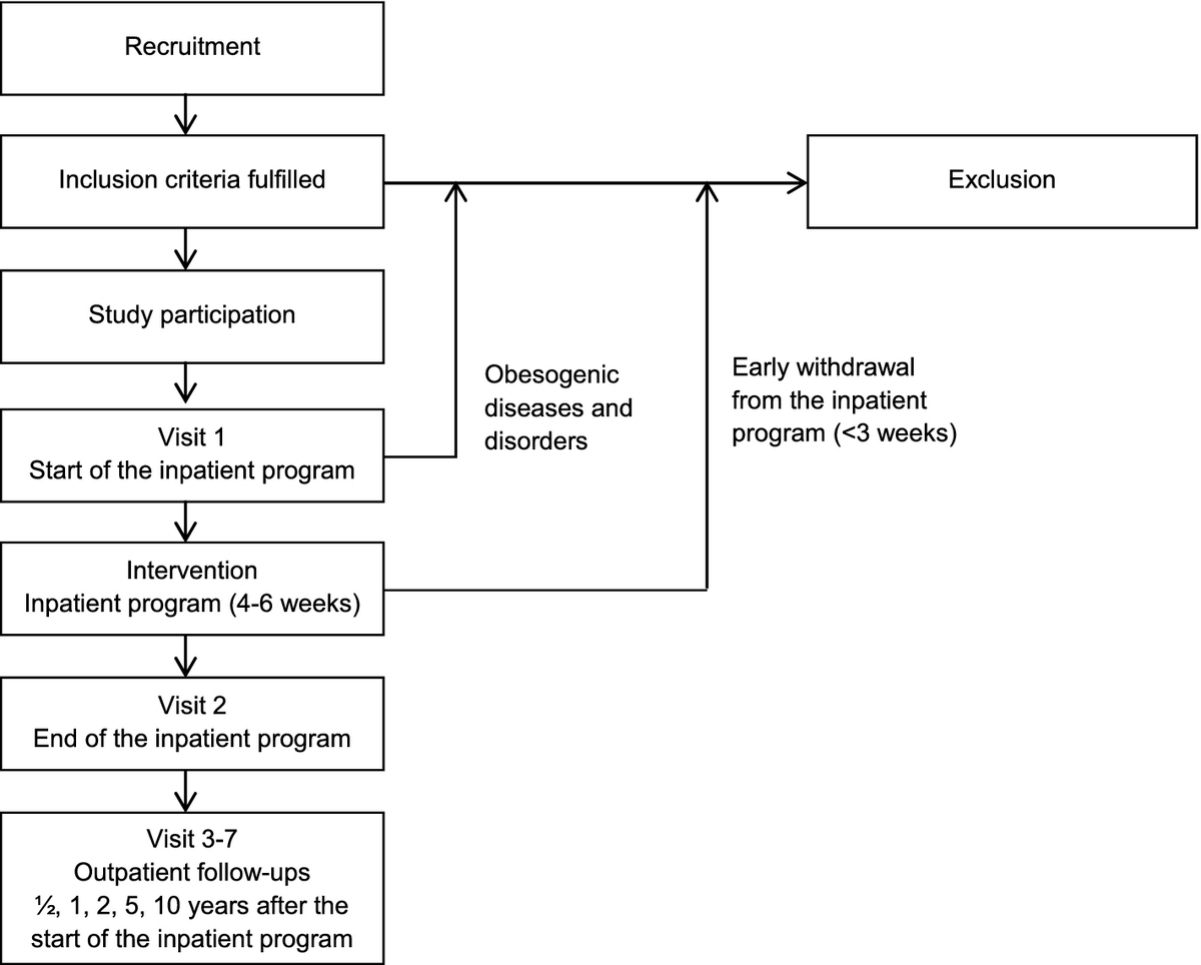
**Study flow chart of the LOGIC-trial**.

Recruitment for this collaborative study began in January 2006 with the aim to include a total of 1,500 participants by 2013. Figure 1 shows the flow chart of the recruitment and the measurement process. Examinations are performed at the start (Visit 1) and at the end of the intervention (generally after 4 to 6 weeks; Visit 2) at the clinic. Follow-up examinations are performed at 6 months (Visit 3), 1 year (Visit 4), 2 years (Visit 5), 5 years (Visit 6) and 10 years (Visit 7) after the start of the intervention by either local pediatricians or general practitioners (Figure 1).

### Intervention

The rehabilitation clinic is primarily focused on inpatient treatment for childhood overweight and obesity which typically lasts for 4 to 6 weeks. The duration of the stay depends on health insurance allowance and the severity of obesity. Typically the children are referred to the clinic for 4 weeks and in case of severe obesity or comorbidities they have the opportunity to extend the program. The standardized multimodal program focuses on a calorie restricted balanced diet, an increase in physical activity and behavioral counseling. It is conducted by an interdisciplinary team of pediatricians, exercise physiologists, dieticians, psychologists and pedagogues according to German guidelines for inpatient weight-loss programs (AGA, Arbeitsgemeinschaft Adipositasim Kindes- und Jugendalter) [31].

The children are offered an optimized balanced diet prepared according to current guidelines (30%, 15% and 55% of the total energy content from fat, proteins and carbohydrates, respectively), with an allowed energy intake of 1,250-1,800 kcal^a ^per day, depending on height and sex (Table 2) [32]. The components of the intervention program are shown in Table 3. In brief, the children are required to participate in theoretical and practical lessons on healthy eating, physical activity and behavior change skills based on the cognitive-behavioral theory. The exercise therapy consists of approximately 10 h of organised physical activity per week,^b ^in addition to 6 hours of recreational exercise.

**Table 2:** Calculation of the allowed energy intake based on body height and sex

Boys		Girls	
Height [cm]	Energy intake per day [kcal]	Height [cm]	Energy intake per day [kcal]
≤ 145	1250	≤ 155	1250
146-170	1500	156-180	1500
≥ 171	1800	≥ 181	1800

**Table 3:** Components of the inpatient weight-loss program

Intervention component	Items	Description	Aims	Frequency/Dose
**Structured physical activity (10 h/week)**	Therapeutic sports	Different types of outdoor activities such as ascending stairs, road running and cross country runs etc.	Endurance training according to individual abilities	2x/week à 60 min
	
	Swimming	Lane swimming ~1.000 m	Endurance training; Learning/improving swimming technique	1x/week à 50 min plus the 6 km walk to the pool (~3 km downhill, 3 km uphill)
	
	Group sports	Different physical activities (e.g. ball games, dancing and gymnastics)	Focus on playing and having fun	1x/week à 45-90 min
	
	Postural training	Strength training: gymnastics, dumbbells, stretch bands, etc.	Strength training to achieve or maintain good posture	1x/week à 45 min
	
	Hiking	10-12 km hikes in the mountains	Endurance training with nature experience	1x/week à 3 h

**Non structured physical activity (~6 h/week)**	'Fun- Walk'	Walking to the town centre (~1 km downhill, 1 km uphill); Time for individual activities	Endurance training, having fun	1x/week à 2 h (in total)
	
	Excursions	Various excursions and activities like playing miniature golf, sightseeing, table tennis tournaments, etc.	Having fun, group activities to improve social skills	Dimension of physical activity varies; within 4 weeks of intervention, it accounts for 6 h/week

**Obesity patient training courses (16 sessions within 4 weeks)**	Psychotherapy	• Developing rules for healthy eating behavior • Rigid versus flexible dieting	Improving self-esteem and body perception, prevention of relapse.	5 session within 4 weeks à 45 min
				
		• Recognition of signs of both hunger and satiety • Learning to enjoy food as well as to cope with difficult situations		
				
		• Developing motivation for participating in regular physical activity		
	
		Individual sessions if the children suffer from psychosomatic, psycho-vegetative and/or psychological diseases	Treating individual psychological problems	1-3 individual sessions à 45 min/week
	
	Nutritional lessons	Teaching children to choose the appropriate (amount of) food according to their personal needs	Enabling the children to prepare healthy food for themselves	5 sessions within 4 weeks à 45 min
	
	Physical education	Improving knowledge on energy balance, effects and limitations of physical activity, measures of self-control and good posture	Increase knowledge of the effects of physical activity to support adherence to the regular physical activity recommendations	4 sessions within 4 weeks à 45 min
	
	Medical education	Improving knowledge on medical background of overweight and obesity (normal/ideal weight, BMI, comorbidities etc.)	Increase knowledge of the medical consequences of overweight and obesity and promote a realistic goal setting	2 sessions within 4 weeks à 45 min

**Social competence**	Training sessions	• Training for conflict resolution, communication, ability to offer and receive criticism, body language, self-assurance, empathy etc. • Role playing • Concentration training	• Development of emotional-cognitive abilities • Development of occupational skills • Reflecting on and improving social behavior skills	1x/week à 45 min

**Nutrition**	Cooking	Cooking as a creative activity and a positive group experience	Transfer of theoretical knowledge into practice	1x/week, 2 h
	
	Lessons for grocery shopping	• Learning how to read packaging labels correctly (e.g. sample sizes, nutritional information) • Learning how to make educated nutritional decisions about potentially misleading products (e.g. 'organic')	Enabling the children to judge different food products correctly	1x/week, 90 min

**Parents**	Supportive training	Parents receive background information on obesity and advice about how to best support their child. In addition, they are requested to take their child to subsequent outpatient psychological treatment. They also receive special handouts about healthy living, including nutrition, physical activity, media consumption etc.	Improving parental support of the children after conclusion of the inpatient program	Two conversations with the physician (at the start and the end of intervention. In special cases, parents are contacted by telephone)

**School**	Theoretical lessons	German, English and Mathematics	Keeping the children current with the appropriate educational curriculum	Groups 3 and 4: 5x/week à 45 min Groups 1 and 2: 6x/week à 45 min

### Measurements

An overview of all measurements at the different time points (Visits 1-7) is presented in Table 4. During the inpatient treatment, the physical examination is performed on the day of admission and on the day of discharge. Blood samples are taken on the third day after admission to the clinic and 3 days before discharge (except for DNA samples, which are taken only at baseline). Physical fitness testing is performed and questionnaires are filled in on the first weekend after admission to the clinic and 1 to 2 days before discharge. In case children extend the treatment, all examinations are being conducted after 6 weeks. Questionnaires are filled in without supervision.

**Table 4:** Overview of the data collection from visit 1 to visit 7

Setting	Inpatient intervention		Outpatient follow-ups			(In/)outpatient follow-ups	
**VISIT**	**VISIT 1**	**VISIT 2**	**VISIT 3**	**VISIT 4**	**VISIT 5**	**VISIT 6**	**VISIT 7**

**Time point**	**Intervention start**	**Intervention end**	**1/2 y**	**1 y**	**2 y**	**5 y**	**10 y**

**Physical examination**							

Anthropometry*	+	+	+	+	+	+	+

Pubertal stage (Tanner)	+		+	+	+	+	+

Comorbidities/Medication	+	+	+	+	+	+	+

**Genetic and blood parameters**							

Collection of EDTA	+						

All blood parameters**	+	+				(+)	(+)

HDL, LDL, total cholesterol, triglycerides, glucose	+	+				+	+

**Physical fitness and activity**							

Physical fitness (ergometry)	+	+				(+)	(+)

6-Minutes running test	+	+				(+)	(+)

Pedometer***	+					(+)	(+)

**Questionnaires (filled in by children)**							

Quality of life (KINDL)	+	+	+	+	+	+	+

Diet/Dietary intake	+	+	+	+	+	+	+

Physical activity	+	+	+	+	+	+	+

**Questionnaire (filled in by parents)**							

Family background	+						

*body weight, body height, waist circumference, blood pressure.

**HDL, LDL, total cholesterol, triglycerides, glucose, proinsulin, insulin, uric acid, TSHbasal, adiponectin, leptin, RBP-4, resistin, high sensitive CRP, IL-6, TNFα.

***subgroup analysis.

#### Physical examination

Body height is measured barefoot to the nearest 0.5 cm by a rigid stadiometer. Body weight is measured with minimal clothing to the nearest 0.1 kg by a digital scale (Visit 1 and 2: *Tanita BC-420 P MA Profi, Tanita Europe B.V*., Hoofddorp, The Netherlands; Visits 3-7: calibrated scale). Waist circumference is measured on bare skin by tape to the nearest 0.1 cm midway between the lower rib margin and the iliac crest in standing position after normal exhalation with a non-stretchable tape measure. Blood pressure is measured at the right brachial artery in the fossa cubitalis after the children have been resting for 5 min in supine position by using a validated protocol [33]. Pubertal development is determined according to Marshall and Tanner [34,35]. Data on the medical history are documented including current medication and comorbidities (orthopedic complications, attention deficit (hyperactivity) disorders, thyroidal diseases, asthma, metabolic diseases, psychological diseases, acute diseases). All inpatient examinations and assessments are conducted by trained medical staff according to standardized procedures.

#### Blood samples

Blood sampling is performed following a 10 hour overnight fast. Samples are taken by venipuncture of an antecubital vein in either a sitting or lying position using vacuum tubes. Both plasma and serum samples are stored at -80°C until analyzed. The following parameters will be analyzed from serum: high density lipoprotein (HDL), low density lipoprotein (LDL), total cholesterol, triglycerides, glucose, proinsulin, insulin, uric acid, TSHbasal, adiponectin, leptin, RBP4, resistin, high sensitive CRP, IL-6 and TNF-α.

#### Genetic analysis

Genomic DNA for all subjects is stored at -20°C after isolation from EDTA blood following a standard protocol. In a first step several SNPs were selected from HapMap CEU data (release 21 phase II, dbSNP 125) including SNPs with minor allele frequencies > 5% in genes of interest for the phenotypes available (e.g. body weight, physical fitness, risk factor profile). In a first step, genotyping was performed using the *MassARRAY *system with *iPLEX™ Gold *chemistry (*Sequenom*, San Diego, CA, USA). The samples were analyzed in a matrix-assisted laser desorption ionisation time of flight mass spectrometer (*MALDI TOF MS, BrukerDaltonik*, Leipzig, Germany). Further analyses will be performed using state of the art genotyping methods.

#### Physical activity and cardiovascular fitness

Physical activity is assessed by a questionnaire and by pedometers. Cardiovascular fitness is assessed by both cycle ergometry and a 6-Minutes running test.

The physical activity questionnaire has been adapted to the MOMO questionnaire, which has been previously validated [36,37]. Items of the questionnaire include volume, frequency, duration and intensity of school, sports clubs and/or leisure time activities, motivation to be physically active [38] as well as questions on sedentary time (screen time and homework). Between 2008 and 2010 all study participants were asked to wear a pedometer (*OMRON Walking Style Pro) *all day for 2 to 4 weeks during their inpatient stay at the clinic. They also completed a physical activity diary for these days.

Exercise testing is performed stepwise on a cycle ergometer (*Jaeger ERGOSTESTER 900*) to the participants' volitional exhaustion. Absolute or relative exercise capacity (Watt, Watt/kg) is used as a measure of cardiovascular fitness. Since 2008 the study participants have been taking part in a 6 min running test. For this test, which takes place on a straight outdoor sports ground, the children are asked to walk or run as far as possible within 6 min. The covered distance is documented in metres.

#### Diet

For the assessment of dietary intake, a food frequency recall is used, which has been validated previously in a survey [39].

#### Quality of life

To assess quality of life, the validated German KINDL^R^-questionnaire [40,41] with six dimensions ("physical well-being", "emotional well-being", "self-esteem", "friends", "family" and "everyday functioning (school)") is being used. The subscales of these six dimensions are combined to a total score. Furthermore, an additional sub-scale, developed specifically to assess the quality of life of overweight children, is being used, which consists of a filter question and six items. The reliability and validity of this questionnaire have been described elsewhere [41].

In addition, a standardized questionnaire that is supposed to be completed by the parents on the day of admission is being used to obtain demographic information as well as obesity-related health history of first degree family members.

### Follow-up (Visits 3 to 7)

#### Visits 3, 4 and 5

Prior to the first follow-up examination, which takes place 6 months after the start of the program (Visit 3), study investigators contact the general practitioners by telephone to inform them about the study procedures and to obtain agreement on carrying out the upcoming follow-up examinations. The general practitioners are asked to complete and return a standardized examination sheet including anthropometric measurements (body weight, height and waist circumference), blood pressure and Tanner stage as well as comorbidities and the current use of medication.

In addition, study investigators contact the children prior to each visit (6 months, 1 year and 2 years after the start of the intervention) to remind them of the upcoming examination and to enquire about possible address changes. The children are requested to complete the questionnaires, previously sent by post, and to return them using the provided prepaid envelope as well as to visit their general practitioner for the follow-up examination. If both the questionnaires and the examination sheet are returned to the study centre, the children will receive an allowance of 10 Euros.

#### Visits 6 and 7

For the 5 and 10 year follow-up examinations, the children are invited to visit the study centre at the *Department of Prevention, Rehabilitation and Sports Medicine, Technical University of Munich*, where the same measurements as at baseline (Visit 1) are planned to be obtained (except for DNA and family history). Children, who are not able to visit the study centre, have blood samples taken by their general practitioner in addition to the basic examination as carried out for the previous follow-up examinations. The blood parameters analysed are fasting HDL, LDL, total cholesterol, triglycerides and glucose. The allowance for each of this visit is 20 Euros.

At all visits, children whose documents have not been returned to the study center are contacted by telephone, repeatedly if necessary, in order to collect the missing documents. If children wish to withdraw from the study in spite of efforts to motivate them to continue participating, study investigators fill out an official drop out sheet.

### Statistical considerations

Associations between polymorphisms in adiposity-relevant genes (*FTO, MC4R, TMEM-18*) and changes in BMI(-SDS) from the start to the end of the intervention, will be assessed using analysis of covariance (ANCOVA) models comparing mean changes in BMI(-SDS) between the two groups of homozygous and the group of heterozygous children adjusted for age, sex and baseline weight. A two-sided level of significance of α = 0.05 will be used. For pairwise group comparisons, two-sample t-tests will be conducted using a Bonferroni-adjusted level of significance of α* = 0.0167.

Middle and long-term associations between genes and measures of interest such as weight change, physical fitness and physical activity will analogously be analysed in an explorative manner. Linear regression models including all relevant genes plus baseline weight, age and sex will be fit into estimate predictive models for the expected short and long-term weight changes. Predictive accuracy of the models and most relevant genes will be assessed using re-sampling methods (e.g. bootstrap) [42]. To estimate the influence of genes on relevant measures over time, a mixed model will be fit to account for multiple measures in the same participants. Missing values will be replaced using multiple imputation methods based on observed values with varying assumptions. Differences in the results obtained by different imputation strategies will be reported and discussed.

With a sample size of 1,500 children the study is sufficiently powered to detect significant differences in all pairwise comparisons between allele groups on an adjusted two-sided level of significance of α* = 0.0167, if the true difference in means is at least half of the common standard deviation translating to an effect size of 0.5 (power > 90% for each pairwise comparison). The sample size calculation is based on the assumption that the distribution of alleles leading to the smallest subgroups will be 70%, 25% and 5%, hence the smallest sample sizes for pairwise comparisons will be 375 versus 75 children. Sample size estimation was conducted for a two-sample t-test with unequal group sizes using the software nQuery (Version 7.0).

## Discussion

This manuscript provides an outline of the rationale and the design of the LOGIC-trial, which is the first study that evaluates the short, middle and long-term effects of an inpatient weight-loss program in association with genetic factors in a large group of children and adolescents (aimed sample size n = 1,500) and includes follow-up measurements over 10 years. Hence, this study will allow the investigation of important determinants of successful weight-loss, particularly the role of a specific genetic predisposition. To achieve this, a large amount of data is being collected, on anthropometry, blood parameters (adipokines and inflammatory markers), physical fitness, physical activity and quality of life.

To our knowledge, only 24 evaluated inpatient programmes have been published, of which merely 14 carried out follow-up assessments. In all studies but one the follow-up periods lasted no longer than three years. No study has ever carried out follow-up measurements after more than five years following an inpatient weight-loss program [18,22-24]. Therefore, our study is unique particularly regarding the 5 and 10 year follow-up measurements and allows investigating the tracking of the effects of an inpatient lifestyle intervention from childhood to adolescence and adulthood. In addition, the large sample size of 1,500 children allows a thorough investigation of the genetic questions of interest. The question of genetic predisposition is particularly interesting regarding obesity and weight change, as obesity is considered as a polygenic syndrome with various SNPs involved. To date, however, the impact of the SNP's on the individual responses to obesity treatment in children is still unclear. The studies that have shown an influence of genetic factors on changes in body weight induced by a lifestyle intervention in children [25-30] had relatively small sample sizes (n = 236 to n = 519) and have shown inconsistent results. A clear advantage of the LOGIC-trial protocol is the inclusion of adipokines and inflammatory markers, as well as objective measures of physical fitness, which will allow investigations of the associations between changes in body weight, inflammation and physical fitness. These investigations are of particular relevance in light of potentially important links between these parameters as indicated by a recent review [7]. Some studies have shown relevant associations between adipokines and weight-loss induced by lifestyle interventions [43-46], whereas particularly the results concerning the associations between adipokines and physical fitness are equivocal. This can be explained by the small sample sizes and different outpatient study settings [47-51]. A further strength of the LOGIC-trial is that all anthropometrical parameters are taken by either a nurse or a general practitioner. This avoids the underestimation of body weight that is often observed in self-reports [52]. The inpatient setting is standardised in that participants are living in a controlled environment with similar dietary and exercise conditions and intervention. Such a controlled setting is particularly important for the investigation of the influence of genetic factors, which can be strongly confounded by environmental conditions [15].

Our study has a few limitations, which cannot be completely avoided in this real-life setting. This is an observational study and not a randomized controlled trial. In a randomized design with a 10 year follow-up time it would be ethically questionable to randomize children into an inpatient weight-loss programme and a control group, as the children from the control group would not be allowed to take part in the lifestyle intervention during that time. In addition, the primary intention of this study is to investigate the inter-individual variability of the effects of the intervention depending on the children's genotypes, which does not necessarily require a control group. For cross-sectional analyses, we use an age-matched sample of normal weight children of a school-based intervention study [53] as well a cohort of young athletes, who are recruited at the *Department of Prevention, Rehabilitation and Sports Medicine, Technical University of Munich*.

As we recruit a selected cohort of children who take part in a specialized obesity program it has to be considered that data from clinical samples may not be representative for general populations. Furthermore, although we do have objective physical activity measurements during the intervention, long-term physical activity is assessed by questionnaires. It has been planned this way as we require a standardised physical activity assessment method that can be carried out by all participants for every follow-up measurement during this 10 year time period. Considering the inclusion of 1,500 children and in total seven measurement time points, objective physical activity measurements would have been almost impossible. Similar to the physical activity, nutritional behavior and intake is assessed by questionnaire. Again, more objective measurements such as dietary records would have been optimal but logistically difficult to integrate. In order to maintain high the compliance of the participants we tried to develop and carry out follow-up examinations that are valid, practical and not too time consuming. Therefore we are not using a detailed food frequency questionnaire.

In summary, this is the first lifestyle intervention study with a detailed assessment of short, middle and long-term weight changes, physical fitness, cardiometabolic risk factors including both inflammatory markers and adipokines in a large cohort of overweight and obese children. Apart from elucidating the short-term effects of this supervised weight-loss program, this study will provide the outstanding opportunity to investigate the tracking of the immediate effects of a lifestyle intervention on body weight and the cardiometabolic risk profile from childhood into adolescence and adulthood under consideration of the influence of genetic predisposition. This will contribute to a better understanding of inter-individual differences in the regulation of body weight and thus may lead to an optimization of personalized treatment strategies for childhood obesity.

## Competing interests

The authors declare that they have no competing interests.

## Authors' contributions

MR has drafted the manuscript. DW has been substantially involved in writing the manuscript. Both are active investigators of the study on site as well as in the analysis center. MH, BW, MS and HL have conducted the study design. In addition, BW was responsible for the design and implementation of the genetic analysis in the study. HL has been coordinator at the *Kinik Schönsicht*. MH, MS, HL, MR and DW have coordinated the study. MH is senior principle investigator. BH has been in charge of the statistical analyses. All authors have critically read and approved the final manuscript. The trial has been registered under clinicaltrials.gov NCT01067157.

## Endnotes

^a^Based on clinic internal considerations this has been changed from 1,200-1,800 to 1,250-1,800 kcal per day in the year 2010.

^b^Based on clinic internal considerations this has been changed from 11 to 10 h per day in the year 2011.

## Pre-publication history

The pre-publication history for this paper can be accessed here:

http://www.biomedcentral.com/1471-2431/12/30/prepub
